# Integrin α3β1 Signaling through MEK/ERK Determines Alternative Polyadenylation of the MMP-9 mRNA Transcript in Immortalized Mouse Keratinocytes

**DOI:** 10.1371/journal.pone.0119539

**Published:** 2015-03-09

**Authors:** Dara S. Missan, Kara Mitchell, Sita Subbaram, C. Michael DiPersio

**Affiliations:** Center for Cell Biology and Cancer Research, Albany Medical College, Albany, New York, United States of America; Thomas Jefferson University, UNITED STATES

## Abstract

Integrin α3β1 is highly expressed in both normal and tumorigenic epidermal keratinocytes where it regulates genes that control cellular function and extracellular matrix remodeling during normal and pathological tissue remodeling processes, including wound healing and development of squamous cell carcinoma (SCC). Previous studies identified a role for α3β1 in immortalized and transformed keratinocytes in the regulation of genes that promote tumorigenesis, invasion, and pro-angiogenic crosstalk to endothelial cells. One such gene, matrix metalloproteinase-9 (MMP-9), is induced by α3β1 through a post-transcriptional mechanism of enhanced mRNA stability. In the current study, we sought to investigate the mechanism through which α3β1 controls MMP-9 mRNA stability. First, we utilized a luciferase reporter assay to show that AU-rich elements (AREs) residing within the 3’-untranslated region (3’-UTR) of the MMP-9 mRNA renders the transcript unstable in a manner that is independent of α3β1. Next, we cloned a truncated variant of the MMP-9 mRNA which is generated through usage of an alternative, upstream polyadenylation signal and lacks the 3’-UTR region containing the destabilizing AREs. Using an RNase protection assay to distinguish “long” (full-length 3’-UTR) and “short” (truncated 3’-UTR) MMP-9 mRNA variants, we demonstrated that the shorter, more stable mRNA that lacks 3’-UTR AREs was preferentially generated in α3β1-expressing keratinocytes compared with α3β1-deficient (i.e., α3-null) keratinocytes. Moreover, we determined that α3β1-dependent alternative polyadenylation was acquired by immortalized keratinocytes, as primary neonatal keratinocytes did not display α3β1-dependent differences in the long and short transcripts. Finally, pharmacological inhibition of the extracellular signal-regulated kinase (ERK)/mitogen-activated protein kinase (MAPK) pathway in α3β1-expressing keratinocytes caused a shift towards long variant expression, while Raf-1-mediated activation of ERK in α3-null keratinocytes dramatically enhanced short variant expression, indicating a role for ERK/MAPK signaling in α3β1-mediated selection of the proximal polyadenylation site. These findings identify a novel mode of integrin α3β1-mediated gene regulation through alternative polyadenylation.

## Introduction

The integrin α3β1 is expressed highly in epithelial cells, where it is the major receptor for laminin-332 and certain other laminin isoform present in basement membranes [1]. α3β1 functions to maintain integrity of the basement membrane during embryonic development of the epidermis, and mutations in the gene that encodes the α3 integrin subunit cause basement membrane rupture and epidermal blistering in both preclinical mouse models and human patients [2–4]. In addition, overexpression of α3β1 occurs in tumors of the epidermis, breast, and other tissues, where it has been linked to promoting tumor growth and progression through the regulation of cell growth, survival, invasion and metastasis, as reviewed elsewhere [5, 6].

Some α3β1-mediated tumor cell functions may be due to its ability to induce the expression of matrix metalloproteinase-9 (MMP-9) [7–9]. Indeed, MMP-9 is an important regulator of tumor angiogenesis and invasion with a prominent role in the development of SCC and other carcinomas [10–12]. Early studies identified mRNA stability as an important post-transcriptional mechanism of MMP-9 gene regulation in response to cytokines, growth factors, and other stimuli [13–15], as well as to integrin α3β1 [16]. Moreover, we previously demonstrated that epidermal keratinocytes acquire α3β1-dependent expression of MMP-9 during immortalization [8, 9]. However, the mechanism whereby α3β1 controls MMP-9 mRNA stability has not been determined.

Post-transcriptional control of mRNA stability is widely documented as an important gene regulatory mechanism in a variety of normal and pathological tissue remodeling processes, including cancer, as reviewed elsewhere [17, 18]. A significant mode of mRNA stability occurs through AU-rich elements (AREs) that reside in the 3’-untranslated region (3’-UTR) and control the rate of mRNA decay [18]. Class I AREs consist of an AUUUA/U pentamer which is often embedded in a uracil-rich region, and are usually present in multiple copies within the 3’-UTR of mRNAs with short or variable half-lives. Generally speaking, ARE-containing mRNAs are labile unless stabilized in response to appropriate extracellular cues. Estimates of the number of human genes that contain AREs range from 5% to 11%, and the presence of AREs is conserved in >50% of mouse and human homologous genes [18–20]. AREs regulate mRNA stability by binding to specific RNA-binding proteins (RBPs). As reviewed extensively elsewhere, examples include AUF-1, isoforms of which have been implicated in both mRNA stability and instability, and members of the ELAV family such as HuR, which normally act to stabilize mRNAs [18]. RBP function can be regulated in response to a variety of stimuli through signals mediated by stress-activated kinases and cell membrane receptors. ARE-mediated control of MMP-9 mRNA stability in response to signals from specific cytokines, nitric oxide, or integrins has been documented [14, 15, 21].

Alternative polyadenylation (APA) is a widely used mechanism of controlling mRNA stability through the generation of alternative mRNA transcripts with distinct 3’-UTR sequences that vary in their ARE content (reviewed in [22]). For example, exclusion of AREs through APA can stabilize a mRNA transcript and lead to higher protein expression. Over half of mRNA-encoding genes in the human genome have multiple polyadenylation [poly(A)] sites, leading to the generation of mRNA variants within the coding regions or 3’-UTR [22]. Importantly, APA as a means to generate truncated 3’-UTRs that lack AREs has been linked to enhanced mRNA stability of pro-tumorigenic genes [23–25]. While some integrins have also been linked to pro-tumorigenic gene expression [26], roles for integrins in controlling APA have not been explored previously.

In the current study, we sought to determine the mechanism through which the integrin α3β1 enhances MMP-9 mRNA stability in immortalized keratinocytes. Initial experiments using standard luciferase reporters for assessing ARE function revealed that the ARE-containing 3’-UTR of MMP-9 confers reduced reporter gene expression whether or not α3β1 is present in the cells, which prompted us to explore whether this integrin instead controls APA that determines ARE content of the mRNA transcript. Indeed, the MMP-9 gene harbors a functional alternative poly(A) site in the 3’-UTR that lies upstream of several AREs [27]. RNase protection assay (RPA) to detect the MMP-9 mRNA variant that is generated by usage of this proximal poly(A) site revealed that it is utilized significantly more in α3β1-expressing than in α3β1-deficient cells. Moreover, we showed that α3β1-dependent APA was acquired by immortalized keratinocytes, as this regulation was not detected in non-immortalized, primary keratinocytes. Finally, we provide evidence that α3β1-dependent APA of the MMP-9 mRNA occurs through a signaling pathway that involves extracellular signal-regulated kinase (ERK)/mitogen-activated protein kinase (MAPK). Together, these data suggest a novel role for integrin α3β1 in immortalized keratinocytes in regulating APA, which in turn determines mRNA stability by controlling ARE content of the 3’-UTR. To our knowledge, these data provide the first evidence that integrins can influence poly(A) site selection, representing a novel mode of integrin-mediated regulation of mRNA stability and gene expression.

## Materials and Methods

### Cell culture

The immortalized mouse keratinocyte (MK) cell lines, MK+/+ (MK-1.16) and MK−/− (MK-5.4.6), were derived from the epidermis of wild type or α3-null mice, respectively, as described previously [8]. MK−/−: hα3 cells were generated from MK−/− cells stably transfected with human α3, as described [16]. Epidermis-specific α3 knockout (α3eKO) mice are homozygous for a floxed α3 allele (α3^flx/flx^) and express Cre recombinase under control of the keratin-14 promoter (K14-Cre), as described [28]. Primary keratinocytes were isolated from α3eKO mice or control littermates lacking K14-Cre using established protocols [8]. Culture conditions for primary keratinocytes and MK cell lines were as described in detail elsewhere [8, 9]. For some experiments, cells were treated for 24 hours with the MEK inhibitor, U0126 (10 μM), prior to analysis.

### Transfection with luciferase reporter plasmids

**Fig 1:**
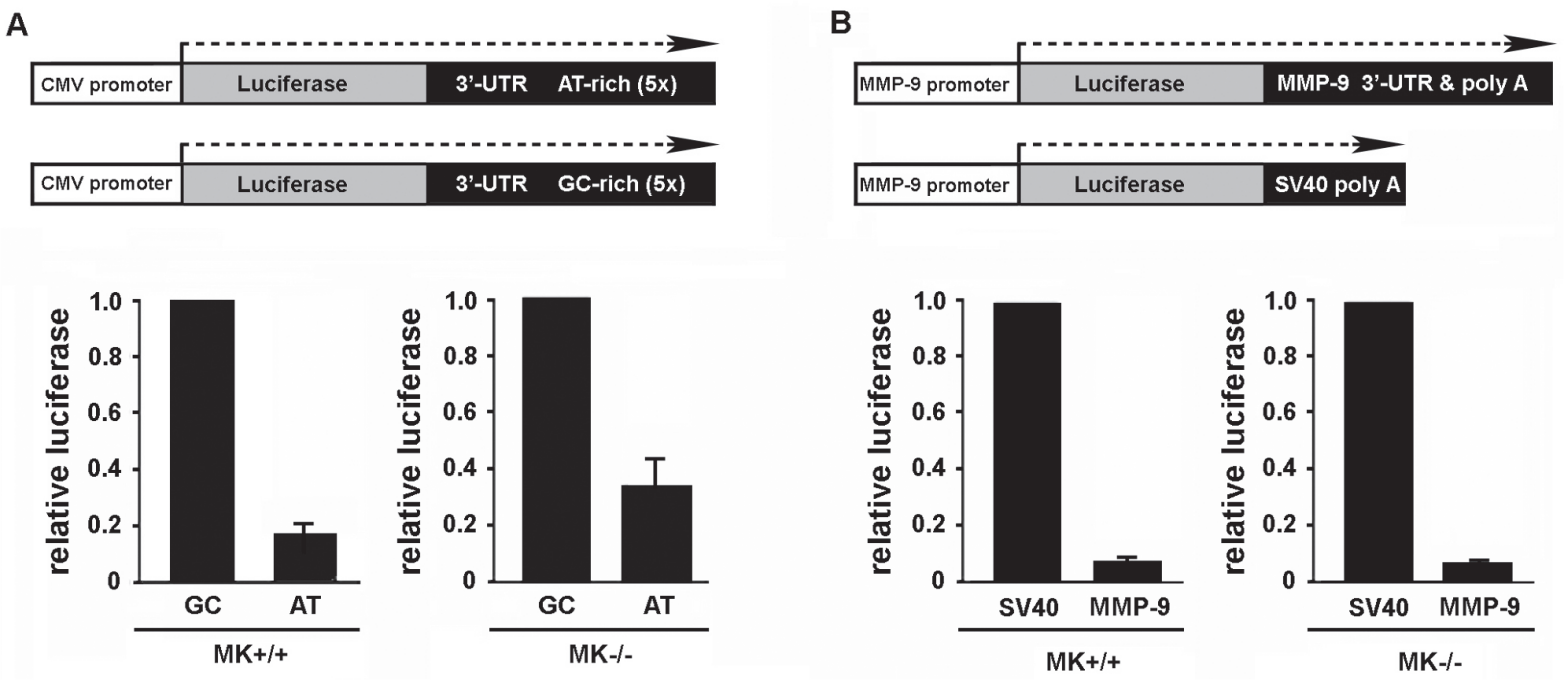
Influence of 3’-UTR AREs on luciferase reporter expression in MK cells that express or lack integrin α3β1. (A) Schematic of CMV promoter-driven luciferase reporter genes containing pentamers that encode either consensus AU-rich ARE sequences or control GC-rich sequences within the 3’-UTR of the mRNA. Reporter plasmids were transfected into α3-expressing MK+/+ cells or α3-null MK−/− cells, and experimental luciferase signals were normalized to that for a co-transfected *Renilla* luciferase control plasmid (pRLTK). Graph shows luciferase activity from the AU-rich reporter relative to that from the GC-rich reporter. (B) Schematic of MMP-9 promoter-driven luciferase reporter genes containing either the MMP-9 3’-UTR or the SV40 poly(A) signal downstream of luciferase coding sequences. MK+/+ cells or MK−/− cells were co-transfected with reporter and control plasmids as in (A). Graph shows luciferase activity from the MMP-9 3’-UTR reporter relative to that from the SV40 poly(A) reporter. For (A) and (B), MK cells were seeded onto LN-332-rich ECM and transfected for 24 hours, then luciferase expression was assayed as described in the Materials and Methods. Data are the mean of three independent experiments +/- s.e.m.

CMV promoter/luciferase reporter plasmids, constructed in the pcDNA3 expression vector (Invitrogen), were generously provided by Dr. Joan Steitz (Yale University). In these plasmids, the 3’-UTR of the firefly luciferase reporter gene was engineered to contain five repeated copies of either a consensus AU-rich ARE (ATTTA), or a control GC-rich sequence, as described [29]. MMP-9 promoter/luciferase reporter plasmids [pGL3-MMP-9(1.3kb)], constructed in the pGL3 Basic Luciferase Reporter Vector (Promega, Madison, WI), were generously provided by Dr. Wolfgang Eberhardt (Goethe-Universität Frankfurt am Main). These plasmids contained either the SV40 poly(A) signal (present in the pGL3 parent vector), or the ARE-containing 3’-UTR from MMP-9 (lacking the proximal poly(A) site) inserted downstream of the firefly luciferase gene, as described [14, 30]. For transient transfections, MK cells were sub-cultured on laminin-332-rich extracellular matrix prepared from the human SCC-25 cell line, as we have described previously [8]. Cells were co-transfected with the above luciferase reporter plasmids and a TK promoter/*Renilla* luciferase internal control plasmid (pRLTK, Promega) at a 50:1 ratio using lipofectamine. After 24 hours, cell lysates were collected and luciferase activity was measured using the Dual-Luciferase Reporter Assay Kit (Promega) and a TD-20/20 luminometer (Turner Designs). For each sample, expression from the experimental luciferase plasmid was normalized to that from pRLTK to control for differences in transfection efficiency, and relative luciferase activity was plotted as described in the Fig. 1 legend.

### Adenoviral infection

Adenovirus expressing a tamoxifen-inducible form of Raf-1 (DRaf-1:ER*) was kindly provided by Dr. Kevin Pumiglia (Albany Medical College), and consisted of a BamH1 fragment encoding tamoxifen-inducible Raf-1 [31] cloned into a Bgl II site in pAdTrack-CMV. Adenoviruses were generated essentially as described previously [32, 33]. MK cells cultured on collagen coated 6-well plates were infected with adenoviral particles plus antennapedia peptide (Anaspec, Fremont, CA) for 24 hours with or without 1μM 4-OH tamoxifen, then cultured for an additional 24 hours (+ or − tamoxifen) prior to preparing RNA or cell lysates, as described below.

### Cloning of MMP-9 3’-UTR

**Fig 2:**
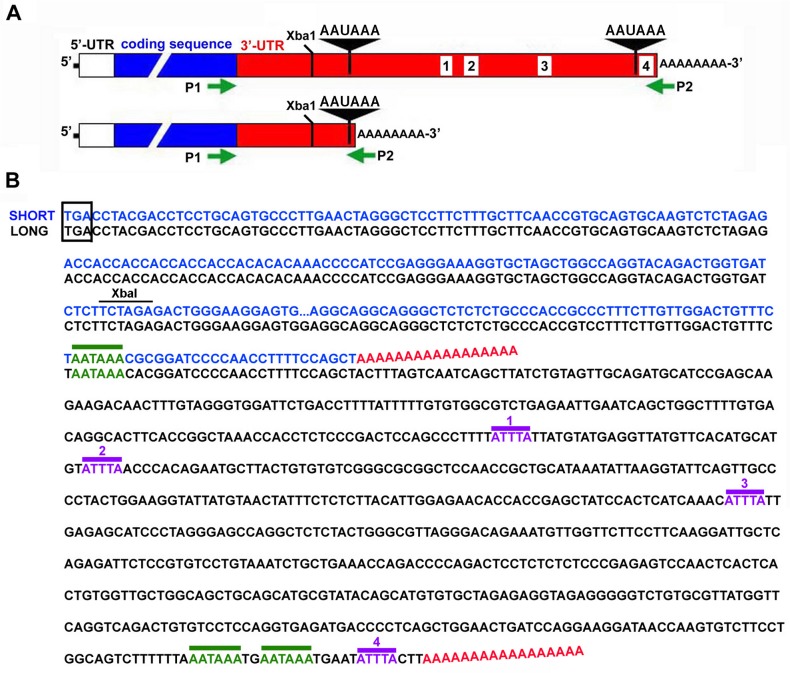
Keratinocytes express a truncated MMP-9 mRNA variant that lacks 3’-UTR AREs. (A) Schematic of MMP-9 mRNA 3’-UTR variants. Sequences corresponding to alternative poly(A) signals (*black triangles)* flank ARE motifs within the long variant (*white boxes*, *1–4*). 5’-UTR (*white*) and protein coding regions (*blue*) are also shown (not to scale). An Xba1 site (for reference) and approximate positions of *P1* and *P2* cloning primers (not to scale) are indicated. (B) 3’-RACE was used to PCR-amplify cDNAs corresponding to the 3’-UTR of the MMP-9 mRNA. The cDNA sequence of a “short” 3’-UTR variant that was amplified from MK+/+ cells (*blue*) is aligned against the known cDNA sequence of the “long” 3’-UTR variant of murine MMP-9 mRNA (*black*) (GenBank: BC046991.1). The stop codon (TGA) adjacent to the 3’-UTR is boxed; the position of polyadenylation for each variant is indicated by the red poly(A); poly(A) signals (AATAAA) are overlined in green (two AATAAA motifs occur near the end of the long variant); four canonical AREs are present only in the long variant and are overlined in purple and numbered 1–4.

A truncated variant of the MMP-9 mRNA that lacks the AREs was cloned from MK+/+ cells using the 3’-RACE System (Invitrogen, Carlsbad, CA) with a forward primer (Fig. 2A, P1) that targets the coding region upstream of the stop codon (5’-GTCTGGATAAGTTGGGTCTAG-3’), and a reverse adaptor primer (Fig. 2A, P2; supplied with the kit) that targets the polyadenylated tails of mRNA transcripts. A PCR product of ∼600 bp was amplified from MK+/+ cells, cloned into the pGEM-T Easy Vector (Promega), and sequenced.

### RNase protection assay

Total RNA was isolated from MK cells using the RNeasy Plus isolation kit (Qiagen,Valencia, CA). RNA was reverse transcribed using the 3’-RACE System (Invitrogen). PCR was performed using Sigma Ready Mix without RedTaq (Sigma, St Louis MO) with a forward primer (5’-CAGGAGTCTGGATAAGTTGG-3’) and the AUAP (abridged universal amplification primer) that binds an adaptor sequence added to the polyA(+) tail from the 3’-RACE kit (5’-GGCCACGCGTCGACTAGTAC-3’). PCR conditions were as follows: 94°C hot start for 3 minutes; denaturation at 94°C for 60 seconds; annealing at 50°C for 60 seconds; extension at 72°C for 120 seconds; 35 amplification cycles with a final extension of 120 seconds at 72°C. Equal quantities of PCR product were assayed using the Ribonuclease Protection Assay kit (Ambion, Grand Island, NY) according to the manufacturer’s protocol. RNA probes were transcribed with biotin-16-UTP using the MAXIscript *in vitro* transcription kit (Ambion). Probes were designed to hybridize either upstream of the proximal poly(A) signal within the 3’-UTR of mouse MMP-9 (control probe, protects 202 nucleotides) or spanning the proximal poly(A) signal (short/long probe, detects 401 nucleotides for long variant, or 241 nucleotides for short variant). Products were electrophoresed on a 5% denaturing urea gel and transferred to Brightstar-Plus membranes (Ambion) using a semi-dry transfer apparatus (Biorad, Hercules CA), at 200 mAmps for 1 hour. Membranes were then UV-crosslinked and assayed using the Brightstart Biodect system for nonisotopic detection (Ambion). Signals were quantified from at least three independent experiments. The MMP-9 mRNA long variant was quantified as a proportion of total MMP-9 mRNA (i.e., summation of the short and long variants), normalized to the daily mean to account for variability by day. For each experiment, data were normalized further by dividing each value by the average value for the control.

### Western blot

Total cell lysates were prepared in non-reducing Cell Lysis Buffer (Cell Signaling Technology, Beverly, MA), and protein concentrations were quantified using the BCA Protein Assay kit (Pierce, Rockford, IL). Equal protein concentrations were subjected to 10% SDS-PAGE, transferred to nitrocellulose membranes, and blocked with 5% BSA/TBST. Rabbit polyclonal antisera against the integrin α3 subunit [34], p-ERK (Cell Signaling Technology), ERK (Santa Cruz Biotechnology, Santa Cruz, CA), and ER (Santa Cruz, Santa Cruz, CA) were used at 1:1000 dilution, followed by horse-radish peroxidase-conjugated goat anti-rabbit IgG at 1:2000 dilution (Cell Signaling Technology). Chemiluminescence was performed using the SuperSignal kit (ThermoScientific, Rockford, IL).

### Ethics Statement

Animal experiments were carried out in strict accordance with the recommendations in the Guide for the Care and Use of Laboratory Animals of the National Institutes of Health. The protocol was approved by the Institutional Animal Care and Use Committee (IACUC) at Albany Medical College (Protocol Number: 12-06004). Euthanasia was performed using carbon dioxide inhalation in a closed chamber followed by death verification by decapitation.

## Results

### mRNA destabilizing AU-rich elements function independently of integrin α3β1

We previously demonstrated that absence of integrin α3β1 in immortalized mouse keratinocytes (MK cells) leads to increased turnover of the MMP-9 mRNA transcript, indicating an important role for this integrin in controlling MMP-9 mRNA stability [16]. The 3’-UTR of the full-length MMP-9 mRNA transcript contains several canonical AREs [27, 35], and previous studies in renal mesangial cells have demonstrated that nitrous oxide and interleukin-1β can alter MMP-9 mRNA stability through regulation of these AREs [14, 15]. To determine whether α3β1 regulates ARE function, we employed a previously established luciferase reporter assay for assessing ARE-mediated effects on mRNA expression [15, 29]. First, we transiently transfected either wild type MK cells (MK+/+ cells) or α3-null MK cells (MK−/− cells) with reporter plasmids in which the CMV promoter drives expression of the firefly luciferase gene to generate a mRNA transcript that harbors pentamers of either consensus ARE sequences or control GC-rich sequences within the 3’-UTR (Fig. 1A). Of note, luciferase reporter signals from the control GC-rich plasmid (normalized to a co-transfected *Renilla* luciferase plasmid) were comparable between MK+/+ cells and MK−/− cells, indicating similar levels of CMV promoter activity in these cells. Thus, any differences in normalized luciferase expression between the AU-rich and GC-rich reporter genes can be attributed to the presence or absence of AREs within the 3’-UTR [29]. For these experiments cells were cultured on laminin-332-rich extracellular matrix, on which they exhibit α3β1-dependent stability of endogenous MMP-9 mRNA [16]. Interestingly, the AU-rich pentamer conferred reduced luciferase expression compared to the GC-rich pentamer in both MK+/+ cells and MK−/− cells (Fig. 1A, graphs), indicating that ARE-mediated reduction of the reporter mRNA occurs independently of α3β1 expression.

To test the possibility that context of the AREs within the MMP-9 3’-UTR is important for α3β1-dependent function, we performed similar experiments using a separate set of luciferase reporter constructs in which either the MMP-9 3’-UTR that encompass the AREs, or the SV40 3’-UTR as a control, was inserted downstream of the reporter gene open reading frame (Fig. 1B) [30]. Again, normalized luciferase signals from the SV40 3’-UTR control plasmid were within ∼1.6-fold between MK+/+ cells and MK−/− cells, indicating similar MMP-9 promoter activity in these cells as we described previously [16]. However, we observed that the MMP-9 3’-UTR conferred reduced luciferase reporter expression, compared to the SV40 3’-UTR control, in both MK+/+ cells and MK−/− cells (Fig. 1B, graphs). Taken together, our results suggest that ARE function in MK cells, either in isolation or within the context of the MMP-9 3’-UTR, is not altered by the presence or absence of α3β1.

### Keratinocytes express a truncated variant of the MMP-9 mRNA that lacks AREs in the 3’-UTR

A review of the literature regarding the cloning and characterization of MMP-9 from various cell types and species revealed the existence of 3’-UTR variants of the MMP-9 mRNA that either contain or lack AREs [14, 27, 35–38]. One early study in which MMP-9 cDNAs were cloned from cutaneous wounds in rats identified two alternative poly(A) signals (AATAAA) in the MMP-9 gene that flank the sequences encoding several AREs in the 3’-UTR [36]. Similarly, examination of the full-length murine MMP-9 cDNA revealed a potential alternative poly(A) signal that lies upstream of four canonical AREs (shown schematically in Fig. 2A, and indicated in the cDNA sequence of the full-length 3’-UTR in Fig. 2B), consistent with a previous report that the MMP-9 gene undergoes APA in murine macrophages [27]. Taken together, these observations suggest that MMP-9 mRNA stability is controlled through generation of transcript variants that contain or lack AREs in the 3’-UTR through alternative use of distal and proximal poly(A) signals, respectively, that flank these elements.

The potential for APA to generate MMP-9 mRNA variants (Fig. 2), combined with our observations that α3β1 expression did not alter ARE function in the reporter assays (Fig. 1), prompted us to explore whether α3β1 promotes use of the proximal poly(A) signal thereby excluding AREs from the 3’-UTR. This model predicts that expression of a shorter variant of the MMP-9 mRNA should be enriched in α3β1-expressing MK+/+ cells, compared with α3β1-deficient MK−/− cells. This prediction was confirmed by 3’-RACE using a forward primer that targets the MMP-9 coding region just upstream of the stop codon (Fig. 2A, P1) and a reverse adaptor primer that targets the polyA(+) tails of mRNAs (Fig. 2A, P2; see Materials and Methods for details). This primer pair is therefore designed to amplify either the full-length or 3’-UTR truncations of the MMP-9 mRNA transcript. Using this approach, we isolated a cDNA from MK+/+ cells that corresponds to a “short” variant of the MMP-9 mRNA. Indeed, alignment of this cloned sequence with the published, full-length 3’-UTR cDNA sequence for murine MMP-9 (i.e. the “long” variant) confirmed that it represents a truncated version of the MMP-9 mRNA transcript that lacks all of the canonical AREs (Fig. 2B). Interestingly, we failed to amplify this short MMP-9 mRNA variant in MK−/− cells using identical 3’-RACE conditions, supporting our hypothesis that this variant is enriched in cells that express α3β1.

### Expression of integrin α3β1 controls the generation of MMP-9 mRNA 3’-UTR variants through use of alternative poly(A) sites that determine ARE content

**Fig 3:**
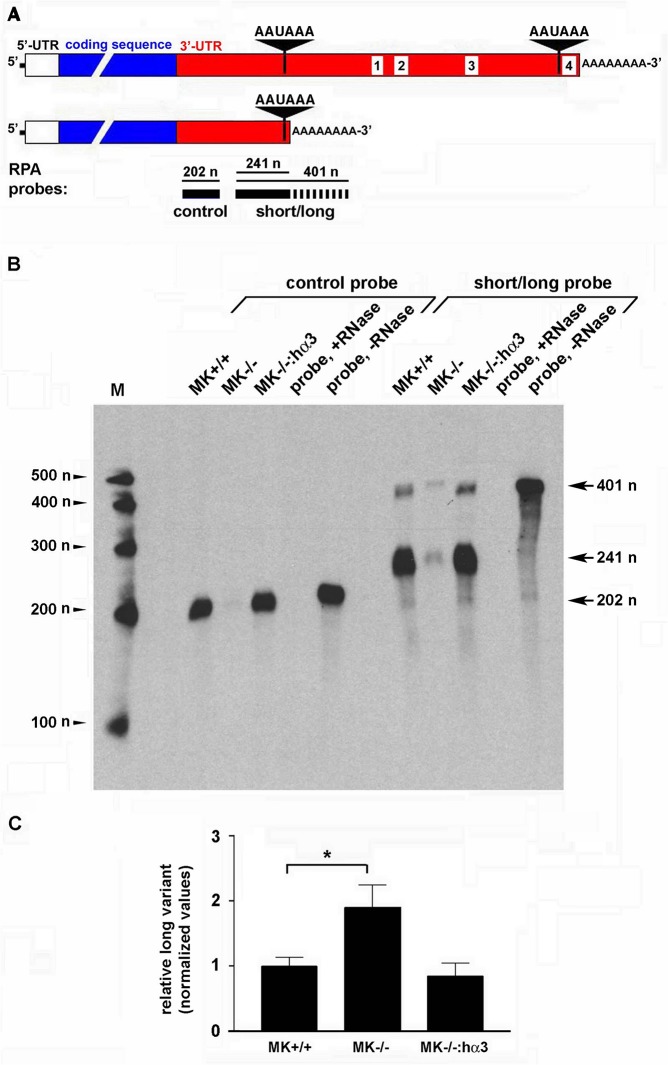
Integrin α3β1 expression enhances MMP-9 mRNA and promotes use of the proximal poly(A) signal. (A) Schematic of RPA probes and their alignment with protected regions of long and short variants of the MMP-9 mRNA. The control RPA probe that corresponds to common 3’-UTR sequences upstream of the proximal poly(A) signal is fully protected by either mRNA variant, while the short/long RPA probe that spans the proximal poly(A) signal is not fully protected by the short mRNA variant (indicated by the dashed portion). Lengths of protected regions are indicated in nucleotides (*n*). (B) RPA was performed as described in the Materials and Methods using the control or short/long probes to assess relative expression of MMP-9 mRNA variants in MK+/+ cells, MK−/− cells, or MK−/−: hα3 cells. Control reactions included probe only in the presence (*probe*, *+RNase*) or absence of RNase (*probe,-RNase*). M, size markers in nucleotides (*n*); lengths of protected probes are indicated to the right. (C) Signal quantification from three independent RPA experiments (see Materials and Methods) reveals that the MMP-9 mRNA long variant constitutes a higher proportion of the total MMP-9 mRNA expressed in MK−/− cells, compared with α3-expressing MK+/+ cells or MK−/−: hα3 cells. Data are mean ± s.e.m. (n = 3); all values were normalized through dividing by the average for the control (MK+/+). *P<0.05, one-way ANOVA, post-test Tukeys multiple comparison.

To directly test our hypothesis that α3β1 influences APA that determines inclusion or exclusion of 3’-UTR AREs, we developed an RNase protection assay (RPA) to detect use of the upstream poly(A) signal and assess the relative amounts of short and long mRNA variants in MK cells that express or lack α3β1. For these experiments, we generated a biotin-UTP-labeled RNA probe that spans sequences corresponding to the proximal poly(A) site and can therefore distinguish use of the proximal and distal poly(A) signals. As depicted schematically in Fig. 3A, this “short/long” probe is only partially protected by a mRNA that is generated through use of the proximal poly(A) signal (indicated by a 241 nucleotide product), but it is fully protected if the proximal poly(A) signal is skipped (indicated by a 401 nucleotide product). As a control, we generated an RNA probe corresponding to a 3’-UTR sequence upstream of the proximal poly(A) signal that is fully protected by either the long or short mRNA variant (indicated by a 202 nucleotide product). Due to very low levels of endogenous MMP-9 mRNA, it was necessary to perform RT-PCR to amplify cDNA products corresponding to the 3’-UTR mRNA variants prior to their detection by RPA (see Materials and Methods). RPA using the control probe revealed that total MMP-9 mRNA levels were markedly reduced in α3-null MK−/− cells compared with wild type MK+/+ cells, but were restored in MK−/− cells stably transfected with human α3 (MK−/−: hα3) (Fig. 3B), consistent with our previous studies showing α3β1-dependent MMP-9 mRNA expression [8, 16]. RPA using the short/long probe detected the short and long mRNA variants in MK cells; as expected, total MMP-9 mRNA (i.e., the sum of short plus long variants) was markedly lower in α3-null MK−/− cells compared with α3β1-expressing MK cells (Fig. 3B). Importantly, however, the relative amount of the long variant as a proportion of the total MMP-9 mRNA was higher in MK−/− cells compared with either MK+/+ or MK−/−: hα3 cells (Fig. 3C). This trend, combined with reduced overall levels of MMP-9 mRNA in MK−/− cells, indicates a shift towards preferential use of the distal poly(A) site when α3β1 is not expressed, presumably leading to a less stable mRNA transcript. These findings suggest that α3β1 promotes use of the proximal poly(A) site to generate the short mRNA variant, leading to exclusion of the 3’-UTR AREs and enhanced stability of the MMP-9 mRNA transcript.

### α3β1-dependent expression of the short MMP-9 mRNA variant is acquired by immortalized keratinocytes

**Fig 4:**
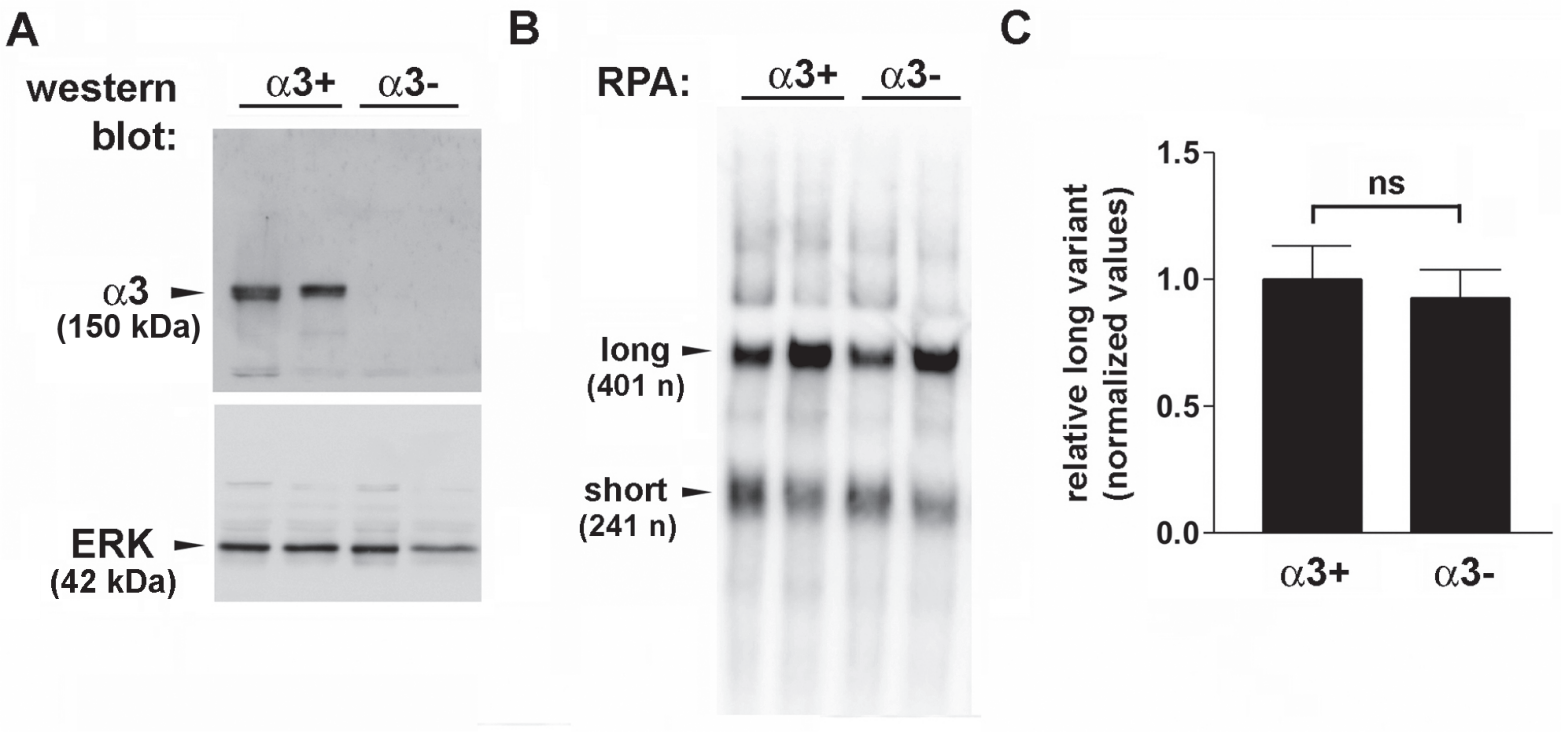
MMP-9 mRNA variant expression is not α3β1-dependent in non-immortalized, primary keratinocytes. (A) Western blot of lysates from representative α3-expressing (*α3+*) and α3-null (*α3−*) primary keratinocytes with antiserum against the integrin α3 subunit, or ERK as a loading control; molecular weights are indicated (kDa). (B) Corresponding RPA of primary keratinocytes using the short/long probe to distinguish MMP-9 mRNA variants. Results shown in (A) and (B) are for two representative mice of each genotype. (C) RPA quantification (performed as in Fig. 3) reveals no significant difference in relative expression of the MMP-9 mRNA long variant between α3-expressing and α3-null primary keratinocytes. Data are mean ± s.e.m. (α3+ keratinocytes, n = 10; α3− keratinocytes, n = 17); all values were normalized through dividing by the average for the control (α3+). Not significant (ns), Student’s two-tailed t-test.

We previously demonstrated that α3β1-dependent regulation of MMP-9 expression is acquired during keratinocyte immortalization, suggesting that this regulation may have important roles in epidermal tumorigenesis [8, 9]. To determine if α3β1-dependent APA was similarly acquired by immortalized MK cells, we isolated primary keratinocytes from neonatal mice that either express α3β1 (control mice) or lack α3β1 in the epidermis (α3eKO mice), as described previously [28, 39], then performed RPA to evaluate expression of MMP-9 mRNA variants. We observed that expression of both the long and short mRNA variants was similar between α3-expressing and α3-null primary keratinocytes (Fig. 4), suggesting that α3β1-dependent MMP-9 mRNA stability in immortalized keratinocytes is due at least partly to the acquisition of α3β1-dependent APA. Interestingly, the long mRNA variant constituted a large proportion of the total MMP-9 mRNA in primary keratinocytes (Fig. 4B), in contrast with immortalized MK+/+ cells which expressed primarily the short variant (Fig. 3B). Although the significance of this difference is not yet clear, it suggests that primary cells from neonatal mice preferentially utilize the distal poly(A) signal. Alternatively, or in addition, the ARE-containing long mRNA variant may be more stable in primary keratinocytes than it is in immortalized MK cells.

### α3β1 promotes APA of the MMP-9 mRNA through a MEK/ERK signaling pathway

**Fig 5:**
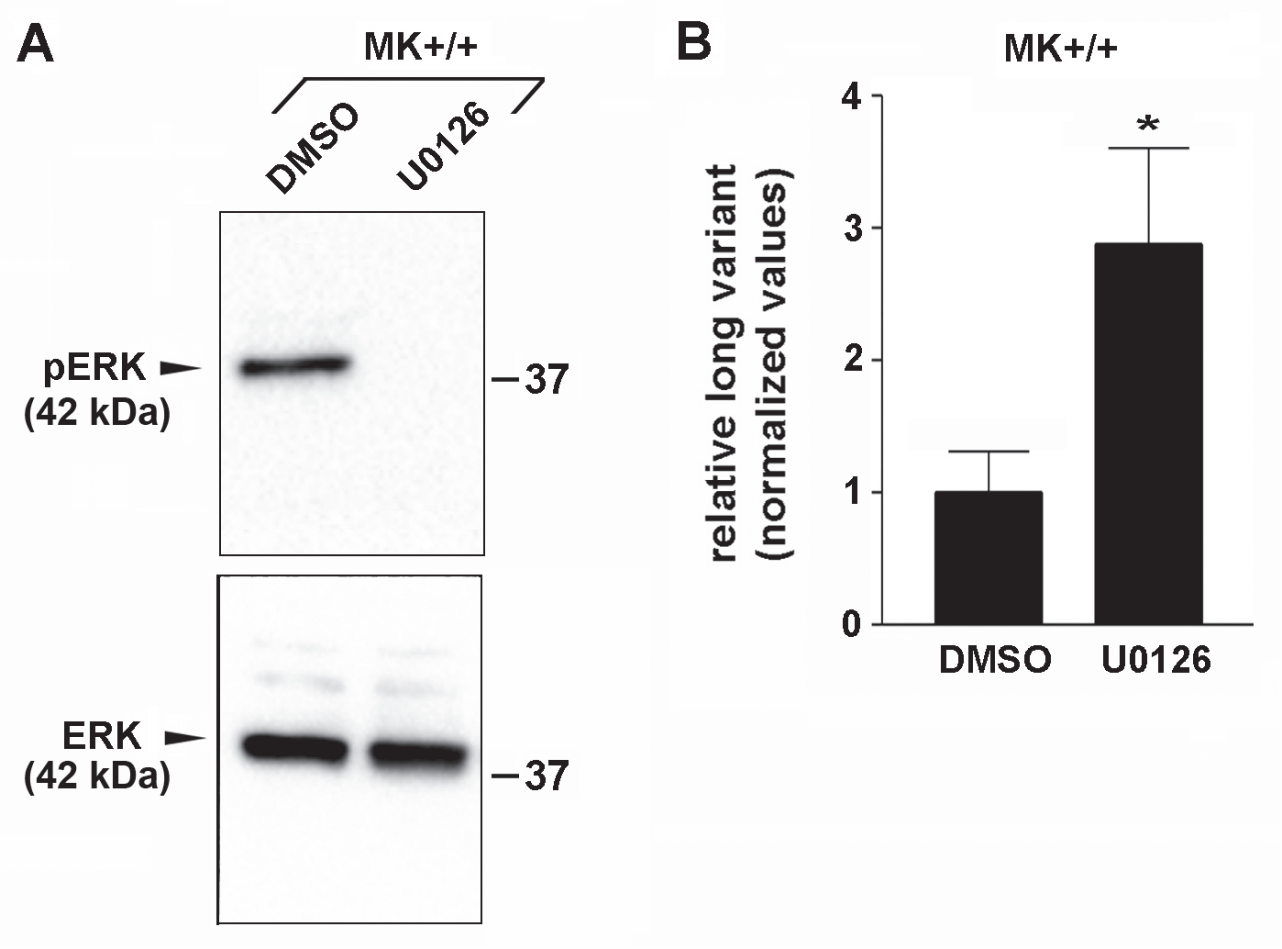
Inhibition of MEK/ERK signaling in α3β1-expressing MK cells increases relative expression of the long MMP-9 mRNA variant. (A) Representative western blot showing inhibition of phospho-ERK (*pERK*) with U0126 compound in MK+/+ cells, compared with DMSO control; *ERK*, total ERK; positions of molecular weight markers are indicated at right (kDa). (B) Quantification of RPA (performed as in Fig. 3) showing the effects of ERK inhibition in MK+/+ cells on the relative amount of the long MMP-9 mRNA variant. Data are mean ± s.e.m. (n = 6); all values were normalized through dividing by the average for the control (DMSO). *P<0.05, Student’s two-tailed t-test.

Previous reports have shown that sustained signaling through the MEK/ERK pathway promotes MMP-9 expression [40, 41]. In earlier studies we observed that α3β1 in MK cells both activates MEK/ERK signaling [42] and induces MMP-9 expression in a MEK/ERK-dependent manner [16], prompting us to test whether MEK/ERK signaling is involved in α3β1-dependent selection of the proximal MMP-9 poly(A) site. α3β1-expressing MK+/+ cells were treated for 24 hours with the MEK1 inhibitor U0126 (10 μM) to inhibit activation of ERK-1/2, or with DMSO as a control, then RPA was performed to compare relative amounts of the short and long MMP-9 mRNA variants. Inhibition of MEK/ERK signaling (Fig. 5A) caused a statistically significant increase in the long MMP-9 mRNA variant (Fig. 5B). Interestingly, treatment of α3-null MK−/− cells with U0126 also enhanced the long MMP-9 mRNA variant, indicating that inhibiting residual ERK signaling observed in these cells [42] similarly reduces proximal poly(A) site selection.

**Fig 6:**
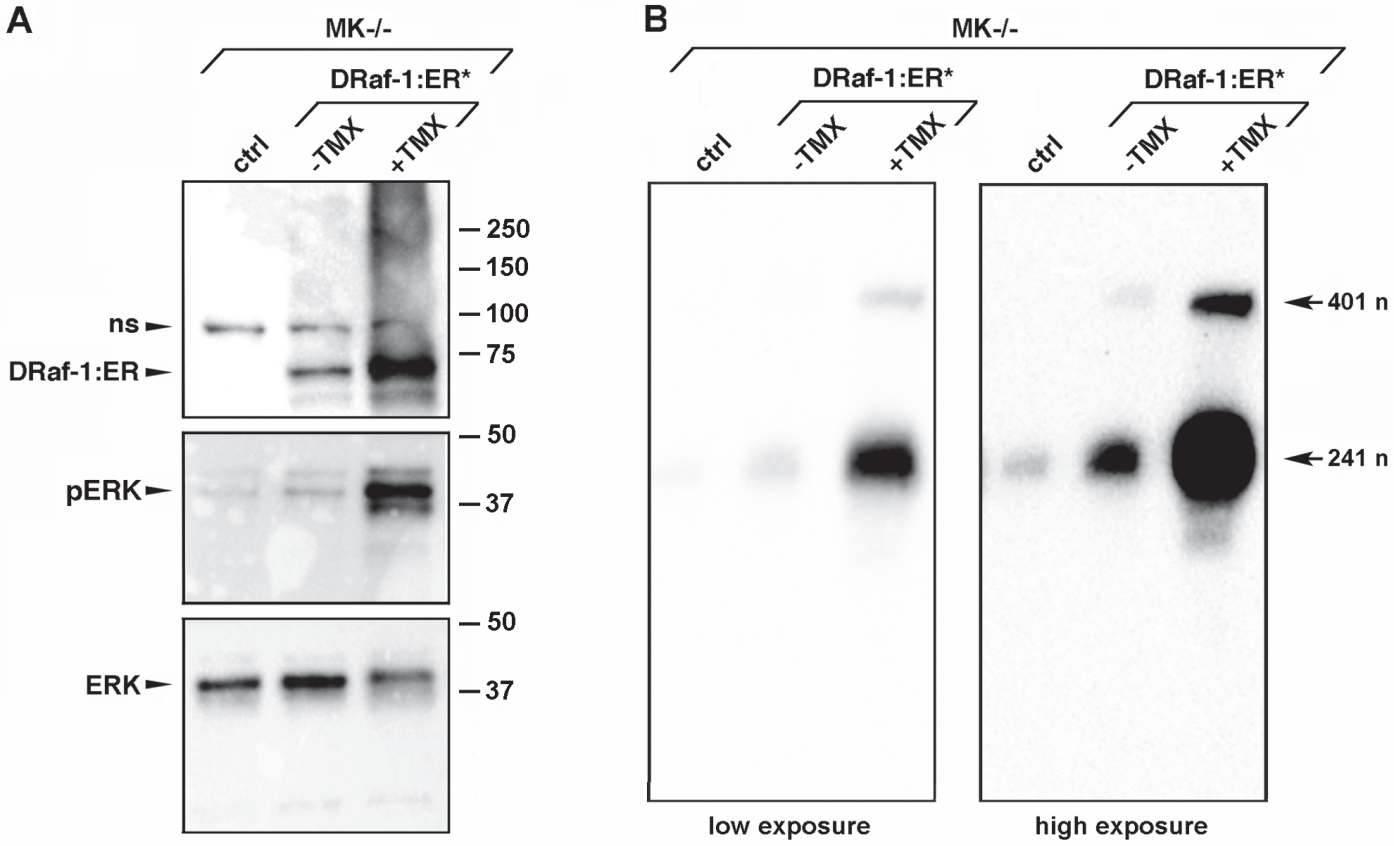
Raf-1-mediated activation of MEK/ERK in α3-null MK cells induces expression of the short MMP-9 mRNA variant. MK−/− cells were left uninfected (*ctrl*) or infected with adenovirus expressing a tamoxifen-inducible form of Raf-1 (*DRaf-1*:ER*) in the absence (*−TMX*) or presence (*+TMX*) of 1 μM tamoxifen. (A) Western blot showing that tamoxifen induction of DRaf-1:ER* leads to enhanced ERK phosphorylation (*pERK*); the latter blot was stripped and reprobed for total ERK (*ERK*); *ns*, non-specific band; positions of molecular weight markers are indicated at right (kDa). (B) RPA showing the effects of basal or tamoxifen-induced Raf-1 on expression of MMP-9 mRNA variants. Two different exposures of the same membrane are shown. Lengths of protected probe are indicated as in Fig. 3. Representative results are shown, n = 3.

To test if enhanced ERK signaling can induce expression of the short MMP-9 mRNA variant in the absence of α3β1, we transduced α3-null MK−/− cells with an adenovirus that expresses a tamoxifen-inducible form of Raf-1 (DRaf-1:ER*) to activate the MEK/ERK pathway, as described [31]. While basal expression of DRaf-1:ER* in the absence of tamoxifen slightly induced ERK phosphorylation in MK−/− cells, tamoxifen treatment of transduced cells caused robust ERK activation (Fig. 6A). RPA of these cells revealed an ERK-mediated increase in both the long and short MMP-9 mRNA variants (Fig. 6B), which could reflect combined inductive effects of ERK signaling on MMP-9 gene transcription and mRNA stability of the ARE-containing long variant (visible in the longer exposure of Fig. 6B). Interestingly, however, we observed that the short mRNA variant was induced to a much higher level than the long mRNA variant (Fig. 6B). This pattern of MMP-9 mRNA variants resembles that seen in α3β1-expressing MK cells (see Fig. 3) and suggests that induction of MMP-9 mRNA in response to Raf-1/MEK/ERK signaling occurs partly through a shift towards generation of the short mRNA. Taken together with our previous studies [16, 42], results in Figs. 5 and 6 suggest that α3β1 signaling through a MEK/ERK pathway enhances MMP-9 mRNA expression in part by promoting use of the proximal poly(A) signal, leading to generation and subsequent accumulation of the more stable short mRNA variant.

## Discussion

**Fig 7:**
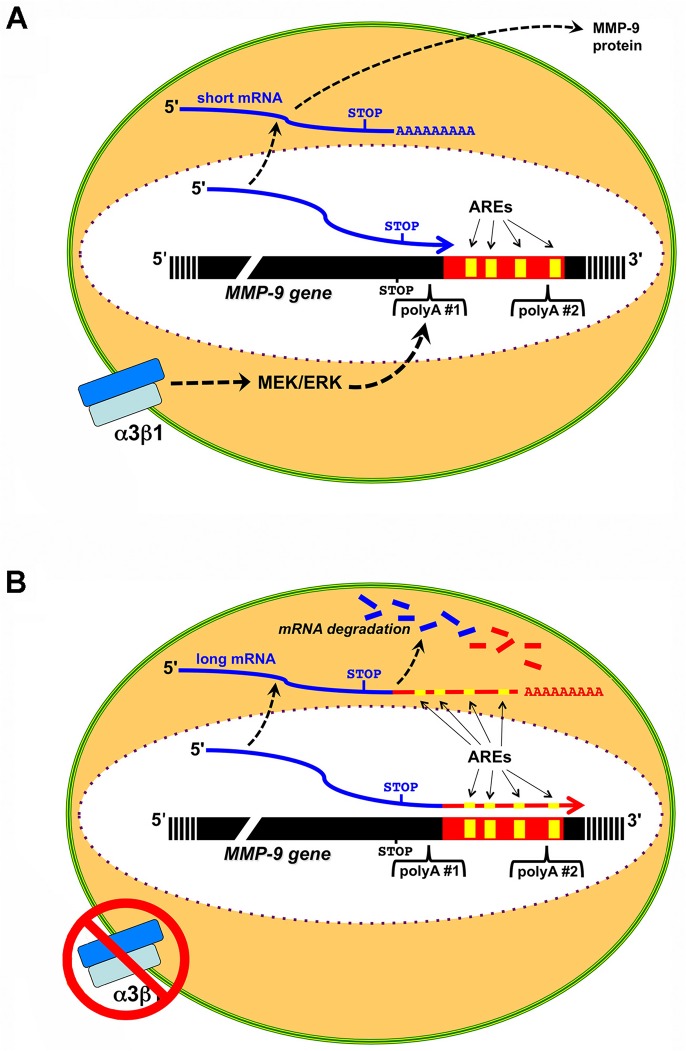
Model depicting α3β1-dependent alternative polyadenylation of the MMP-9 mRNA. (A) Integrin α3β1 activates a MEK/ERK signaling pathway that promotes selection of the proximal polyadenylation site (polyA #1) within the MMP-9 gene, thereby generating the short, more stable mRNA transcript and subsequent synthesis of MMP-9 protein. (B) In the absence of α3β1, MEK/ERK signaling is diminished and polyadenylation defaults to the distal polyadenylation site (polyA #2), thereby generating the long, ARE-containing transcript that is subject to mRNA degradation. Positions of the stop codon, AREs (yellow boxes), poly(A) signals, and poly(A+) tails are indicated.

Alternative poly(A) site usage has emerged in recent years as an important mechanism for regulating the 3’-UTR content of mRNA transcripts, including the inclusion or exclusion of AREs that control mRNA stability (reviewed in [22, 43]). Although some integrins have long been known to regulate gene expression through mRNA stability [16, 21], roles for integrins in controlling APA as a potential mechanism to regulate mRNA stability have been unexplored. In the current study, we cloned a short variant of the MMP-9 mRNA from immortalized keratinocytes that is generated through APA and lacks the 3’-UTR AREs. Moreover, we used an RPA approach to demonstrate that the MMP-9 mRNA pool from cells that express integrin α3β1 is enriched for the short variant, compared with α3-null cells, indicating a novel role for α3β1 in promoting APA that determines the exclusion of AREs from the 3’-UTR. These findings support a model, outlined in Fig. 7, wherein α3β1-mediated MMP-9 mRNA stability that we described previously [16] is controlled through generation of a short 3’-UTR variant that lacks AREs, due to preferential usage of a proximal poly(A) signal that lies upstream of these elements. Thus, our study provides initial evidence that an integrin can regulate poly(A) site selection, representing a new mode of integrin-mediated gene regulation. It will be interesting in future studies to determine whether other integrins can similarly regulate APA, or whether this regulation is specific to integrin α3β1.

It has long been known that neoplastic transformation can stabilize ARE-containing mRNAs [44], and that increased mRNA stability has relevance to genes involved in human cancers [45–47]. However, our current results show that ARE function was not directly altered in immortalized keratinocytes by the presence or absence of α3β1, at least in the context of exogenous ARE reporter constructs. Instead, our results support a role for α3β1 in APA as an alternate means to control mRNA stability by determining ARE content of the target transcript. These observations expand on a novel role that we recently described for α3β1 in controlling post-transcriptional mRNA processing in breast cancer cells, where we showed that RNAi-mediated suppression of α3 promotes alternative splicing of the cyclooxygenase-2 (COX-2) mRNA that targets it for nonsense-mediated decay [48]. Together, these studies point towards a broad role for α3β1 in controlling mRNA stability by regulating the sequence content of target transcripts.

We previously showed that α3β1 in MK cells promotes both MEK/ERK signaling [42] and MMP-9 gene expression [16]. Here we showed that ERK signaling was both necessary in wild type MK cells (Fig. 5) and sufficient in α3-null MK−/− cells (Fig. 6) to promote expression of the short mRNA variant that is generated through selection of the proximal poly(A) site within the MMP-9 gene, suggesting that α3β1-mediated signaling through a MEK/ERK pathway controls APA of the MMP-9 mRNA. It is possible that an ERK-induced poly(A) switch occurs as a result of enhanced MMP-9 gene transcription, since generation of shorter mRNA transcripts through use of upstream poly(A) sites has been linked to enhanced transcriptional activity (see below). Activation of MEK/ERK signaling also enhanced expression of the long mRNA variant (Fig. 6), consistent with previous studies that have linked MEK/ERK signaling to ARE-mediated mRNA stability [49, 50]. Interestingly, signaling via MEK/ERK has been shown to increase MMP-9 production in a range of cancer types, including SCC [51], chondrosarcoma [52], breast cancer [53], fibrosarcoma [54], prostate cancer [55], osteosarcoma [56], and glioma [57], raising the possibility that MEK/ERK-mediated APA plays a role in controlling MMP-9 expression in different types of tumors, possibly in response to different stimuli.

APA is a widely used mechanism of post-transcriptional gene regulation during both normal and pathological tissue remodeling processes [22, 58]. Generation of longer ARE-containing mRNAs may be important for cellular adaptations that require more rapid or regulated changes in MMP-9 mRNA turnover. Indeed, progressive lengthening of 3'-UTRs through APA occurs during mouse embryonic development, perhaps reflecting a need for rapid changes in gene expression that are achieved through incorporation of AREs into the 3’-UTR [59]. On the other hand, reduced 3'-UTR length accompanied by enhanced mRNA stability is often associated with increased cell proliferation [60] and has been linked to oncogene activation in cancer cells [25]. Consistently, we observed that the long MMP-9 mRNA variant was present at a relatively higher level than the short variant in neonatal primary keratinocytes (Fig. 4), while the short mRNA variant was by far the more abundant form in immortalized MK cells that express α3β1 (Fig. 3). These different patterns might reflect preferential use of the distal poly(A) site during perinatal development, and an α3β1-dependent switch towards use of the proximal poly(A) site in immortalized cells. Alternatively, or in addition, the long ARE-containing variant may be more stable in neonatal keratinocytes. Further experiments will be required to distinguish these possibilities.

Genes that are regulated through APA fall into a wide range of functional groups and include transcriptional regulators, intracellular enzymes, extracellular proteases, growth factors and cytokines, integrins, and even RBPs that control ARE-mediated mRNA stability [27, 61–65]. Indeed, one report showed that HuR regulates APA of its own gene to promote ARE inclusion and subsequent down-regulation of HuR mRNA, indicating a negative feedback loop through which HuR auto-regulates mRNA stability [62]. Although mechanisms that control APA remain unclear, analyses of both the human and mouse transcriptomes indicated that shorter 3’-UTR variants are relatively more abundant for genes that are expressed at high levels, while longer 3’-UTR variants are relatively more abundant for genes that are expressed at low levels, suggesting that poly(A) site usage may be coupled with transcriptional activity [66]. Interestingly, our previous studies showed that activity of a transfected MMP-9 promoter was comparable in immortalized MK+/+ and MK−/− cells [16], but it was reduced considerably in these cells compared with non-immortalized, primary keratinocytes [9]. It remains to be determined whether the endogenous MMP-9 promoter shows similar transcription patterns in primary versus immortalized cells. However, since α3β1-dependent regulation of the MMP-9 gene was acquired by immortalized keratinocytes [9], it is possible that α3β1 becomes required in these cells to promote proximal poly(A) site usage as transcription is reduced, in order to maintain sufficiently high MMP-9 mRNA levels through enhanced stability.

Integrin α3β1 has important roles in promoting the growth of epidermal tumors [9, 67]. Many pro-tumorigenic functions of α3β1 may be controlled through lateral association with the tetraspanin family member, CD151, which occurs within tetraspanin-enriched microdomains on the cell surface [68–70]. Indeed, others have shown that CD151 promotes tumor growth and metastasis [71, 72], and in some cases the CD151/α3β1 complex has been shown to regulate expression or proteolytic activity of certain MMPs [73, 74]. Interestingly, CD151 can promote MMP-9 expression in some tumor cells [75], and RNAi-mediated suppression of CD151 in epidermal carcinoma cells led to the internalization of α3β1 accompanied by down-regulation of MMP-9 [76]. These previous findings raise the intriguing possibility that the CD151/α3β1 complex plays a role in the regulation of MMP-9 mRNA stability. However, it remains to be determined whether α3β1 must interact with CD151 to promote APA or stability of the MMP-9 mRNA in our keratinocyte model, and this interesting question will be the subject of future studies.

In a recent genome-wide comparison of wild type and α3-null keratinocytes, we showed that α3β1 regulates numerous genes with potential roles in extracellular matrix remodeling and tumor growth/progression [39]. Interestingly, for the majority of these genes α3β1-dependent regulation was acquired in immortalized cells (i.e., it was not observed in non-immortalized primary keratinocytes), as we had also reported for MMP-9 [8, 9]. Our current observation that α3β1-dependent APA of the MMP-9 gene was similarly associated with immortalization suggests that this mode of regulation contributes to the acquisition by immortalized cells of α3β1-dependent MMP-9 expression. Thus, α3β1-dependent APA may reflect an adaptation that occurs in tumorigenic cells to promote MMP-9 expression, thereby driving tumor growth and progression. This regulation could extend to other pro-tumorigenic/pro-angiogenic genes that are regulated by α3β1, including fibulin-2 and MRP3 in MK cells [28, 39], and COX-2 in breast cancer cells [48], as these genes harbor potential APA sites that in some cases flank canonical AREs within the 3’-UTR [65, 77]. It will be interesting to determine whether α3β1 similarly regulates APA of these or other target genes, and if so whether this regulation is acquired by immortalized or transformed cells as it is for MMP-9. Further characterization of the pathways that control α3β1-mediated APA may reveal novel vulnerabilities that are acquired by tumor cells and can be exploited as therapeutic targets to alter cancer-promoting gene expression programs without affecting gene regulation in normal cells.
